# External quality assessment of AFB smear microscopy performances and its associated factors in selected private health facilities in Addis Ababa, Ethiopia

**DOI:** 10.11604/pamj.2016.24.125.7459

**Published:** 2016-06-09

**Authors:** Lemi Mosissa, Abebaw Kebede, Tedla Mindaye, Muluwork Getahun, Sisay Tulu, Kassu Desta

**Affiliations:** 1 Addis Abeba University, College of Health Sciences, School of Allied Health Sciences Department of Medical Laboratory Sciences, Addis Abeba, Ethiopia; 2 City Government of Addis Ababa Technical & Vocational Education & Training (TVET) Agency, Addis Abeba, Ethiopia; 3 Ethiopian Public Health Institute (EPHI), Addis Abeba, Ethiopia; 4 Minilik II Referral Hospital, Addis Abeba, Ethiopia

**Keywords:** EQA, panel testing, onsite evaluation, blinded rechecking, major error, minor error

## Abstract

Tuberculosis (TB) is still a public health problem in sub Saharan African countries. In resource-limited settings, TB diagnosis relies on sputum smear microscopy, with low and variable sensitivities, especially in paucibacillary pediatric and HIV-associated TB patients. Tuberculosis microscopy centers have several weaknesses like overworking, insufficiently trained personnel, inconsistent reagent supplies, and poorly maintained equipments; thus, there is a critical need for investments in laboratory infrastructure, capacity building, and quality assurance schemes. The performance of TB microscopy centers in the private health facilities in Addis Ababa is not known so far. The main objective of the study was to assess laboratory performance of acid fast bacilli (AFB) smear microscopy and its associated factors in selected private health facilities in Addis Ababa, Ethiopia. A cross-sectional study was conducted in 33 selected private health facilities of Addis Ababa, Ethiopia comprising 7 hospitals, 2 NGO health centers, 23 higher clinics and 1 diagnostic laboratory that provide AFB smear microscopy services. The study was conducted from January to April 2014. A total of 283 stained sputum smears were randomly collected from participant laboratories for blinded rechecking, 320 panel slides were sent to 32 microscopy centers to evaluate their performance on AFB reading, staining and reporting. Checklists were used to assess quality issues of laboratories. Data were captured, cleaned, and analyzed using SPSS version 16.0; χ^2^ tests, kappa statistics were used for comparison purpose. P value < 0.05 considered statistically significant. Among the 32 participant laboratories, 2-scored 100%, 15 scored 80-95% & the remaining 15 scored 50-75% for overall proficiency test performance. There were 10 (3.15%) major errors and 121 (37.8%) minor errors. The sensitivity, specificity, PPV and NPV of panel reading by microscopy centers were 89%, 96%, 96%, and 90% respectively. Out of 283 randomly selected slides for blind rechecking, 11 (3.9%) slides interpreted falsely for AFB, with overall agreement of 97.5%, sensitivity of 88.4% and specificity of 99.3%. In terms of slide quality assessment, 71.6% of AFB slides were graded as good for evenness, cleanness, thickness, size, staining and labeling. The performance score for AFB slide evenness was 56.9% (161 slides) and for labeling quality was 90.8% (257 slides); having significant difference in slide quality (p value < 0.05). On-site evaluation indicated problems in terms of infrastructure, standard operating procedure, reagent quality; equipment maintenance, data management and training issues. Most of the health facilities had poor maintenance scheme for microscope (53.5%) and poor inventory management (25.0%) system. Microscopy centers that scored a proficiency of 75.5%; which is below the acceptable minimum score of 80% and an overall error rate of 3.9% for blinded rechecking needs attention. Moreover, there are gaps identified through on site assessment including poor SOP, reagent quality, equipment maintenance, data management & lack of updated training on AFB microscopy techniques, requiring a concerted effort to alleviate the bottle neck problems and strengthening the public private partnership to control TB.

## Introduction

Tuberculosis (TB) is an infectious and transmissible disease caused by *M. tuberculosis* and occasionally by *M.bovis*; the main pathogenic species within the M. tuberculosis complex. It remains a major cause of human morbidity and mortality, with an estimated 9 million new cases and 1.3 million deaths per year, with most cases occurring in low-income countries [1]. In resource-limited countries, sputum smear microscopy is the routine and available diagnostic method. The technique is simple, inexpensive, and efficient in detecting those cases of pulmonary tuberculosis that are most infectious. Since its yield is highly dependent on execution of the laboratory personnel or even auxiliary, personnel performing smear. Smears may be negative or may require even more careful screening to identify low numbers of AFB [2]. If the laboratory results of AFB are unreliable, then patients with infectious TB may not be diagnosed, resulting in ongoing transmission of disease in the community and more severe disease in the individual. Alternatively, patients without TB may be treated unnecessarily. Therefore, quality assurance of AFB smear microscopy is essential [3]. Accuracy and reliability of laboratory testing are critical to the success of TB control programs. It must be monitored to ensure the quality of the overall process, to detect and reduce errors, and to improve consistency between testing sites. The minimum number of AFB necessary to produce a positive smear result has been estimated to be 5000-10,000/ml of sputum. If AFB concentration is below 1000 bacilli per ml of sputum, the chance of observing the bacilli in a smear is less than 10% [4].

Tuberculosis control strategy needs to be integrated with clinicians, laboratorians, and TB-control officials and effective public-private partnerships that require prompt, complete, and accurate communication among the laboratory systems [5]. Quality assurance of sputum microscopy includes laboratory arrangement & administration, equipment, specimens and request forms, reagents, stains, staining, smear examination and reporting. Sputum microscopy is recommended in current TB control strategies due to its attractive technology for public-health programs, provides visual evidence of TB's bacterial burden, is specific enough that no confirmatory testing is needed, only tiny amounts of material are examined, as little as 0.2 ml, hence bacteria must be presented in high concentrations to be visible, typically over 10,000 AFB per ml, takes about 3-5 minutes [6]. Onsite evaluation allows observation of worker performance under actual working environment, including condition of equipment, laboratory safety, and adequacy of supplies, specimen collection, and the process for smearing, staining, reading, recording, and reporting of stained smears. When problems are detected, solutions can be suggested and potentially implemented immediately [7]. Previous work in Ethiopia focusing in public laboratories showed that, the performance of laboratories were poor in terms of reading slides and unsatisfactory reporting (below acceptable score < 80%) and major & minor errors were identified [8]. Information concerning the performance of AFB microscopy centers from the private health facilities in Addis Ababa is very scarce; hence we planned to determine the performance of the selected private laboratories using the three types of external AFB quality assessment schemes.

## Methods

**Study design and setting:** Cross-sectional study was conducted in Addis Ababa private health facilities that provide AFB smear microscopy diagnostic services.

**Sample size determination:** According to health and health-related indicators released from federal ministry of health there are 35 private hospitals in Addis Ababa, among these 11 are on direct observed treatment (DOT) program and a report from Addis Ababa health bureau, 25 of them provide anti-TB drugs officially. We used the standard sample size determination method with the formula n = z (a/2)^2^ p (1-p)/ d^2^ and n was 384, and from this the finite population correction factor was used to reach on the final sample size. Since the calculated sample size from the above formula was 384, the total population was 36 (11+25); therefore, using the finite population correction factor to estimate final sample size (nf) from target population (N), the sample size was reduced to (Nf= n/1+n/N, nf= 384/1+384/36, 384/ (420/36) = 384/11.66, nf = 33) 33. Hence, the final sample size includes 33 hospitals and clinics. Sample size for interview of the health facilities was calculated with the formula nf= n/ (1+n/N), nf= 173/(1+173/384)→173/(557/384)→173/1.45= 119→ nf = 119, When 10% non-response rates was considered, the sample size calculation were 119+12 (10% non-response rate); 131 based on the above correction method, the minimum sample size was 131.

### Data collection

**Panel testing:** A set of 10 slides were considered as acceptable number according to the Ethiopian national reference laboratory guidelines for QA of smear microscopy for TB diagnosis; this set of slides consisted of five stained and five pre-fixed unstained smears. For this study, 330 validated panel-testing slides including negative (165) and positives (165) of different grade 3^+^→33, 2^+^→33, 1^+^→33, and scanty slides (actual no. of AFB) →66 respectively. Five stained and five unstained slides were randomly selected from each group, the latter was stained, read and quantified by the participant laboratories. While the stained slides panels were directly examined and interpreted according to the SOP. Multiple 3+ smears were not included. Smears were evaluated for appropriate size, thickness, staining quality, cleanness, and evenness as well as labeling by a group of senior laboratory technologist before dispatch. All routinely examined slides (283 processed slides) from January to April 2014 were rechecked onsite by experienced third person and the discordant slides were brought to the national reference laboratory for resolving the problems. Scores for grading used a set of 10 panel testing slides, each slide carries 10 points, and total possible score was 100 (for 10 slides). Committing major error (HFP and HFN) results in a score of 0 where as minor error (LFP, LFN and quantification errors, QE) result in a score of 5 points. Passing score was 80 points and above.

**Data analysis:** Data were entered and analyzed using SPSS version 16.0 and results were explained using absolute numbers, and percentages. Kappa statistics were calculated to show the association between microscopy centers and the NRL as well as inter-laboratory comparability. Besides this, we calculated sensitivity, specificity PPV and NPV to show the test accuracy. We also calculated the χ^2^ to see the association between different factors and significant level was taken at p value less than 0.05. Ethical consideration The ethical clearance was obtained from Addis Ababa University, College of Health Sciences, Department of Medical Laboratory Sciences Research and Ethical Review Committee (DRERC), Addis Ababa Regional Heath Bureau Ethical Clearance Committee and the Ethiopian Public Health Institute (EPHI) Scientific and Ethical Review Office (SERO). Moreover, participant laboratory personnel and the health facilities agreed to participate in this study. We kept their privacy and confidentiality throughout the study. Feedbacks were given for each microscopy centers.

**Limitation of the study:** Annual slide positivity rate was not calculated due to lack of stored data of previous performances at all MCs; We were unable to include all health facilities in the region for better evaluation of the microscopy centers; The blinded rechecking we did was only for one quarter it would be better if four quarters for further elucidation.

## Results

### Participant microscopy centers

In this study, 33 private health facilities were included comprising 7 (21.2%) general hospital, 2 (6.06%) NGO health centers, 23 (69.7%) were higher clinics and 1 (3.03%) diagnostic laboratory (Table 1). All laboratories were willing to participate in the study except one higher clinic and three hospitals that refused to admit the research project for unknown reason and later substituted by higher clinics.

**Table 1:** Comparison of stained & unstained panel-testing of PHF in Addis Ababa, 2014 (n, 32)

Types of panel slides	Major error		Minor error			Total error
	HFN	HFP	LFN	LFP	QE	
Stained panel slide	3 (0.9%)	1 (0.3%)	20 (6.3%)	0	34 (10.6%)	58 (18.1%)
Unstained slide	6 (1.9%)	0	25 (7.8%)	5 (1.6%)	37 (11.6%)	73 (22.8%)
Total	9 (2.8%)	1 (0.3%)	45 (14%)	5 (1.6%)	71 (22.2%)	131 (40.9%)

### Panel testing

A total of 330 AFB smears of various grades of slides were used. The response rates of participant laboratories were 97% as one health institution refused to read the panel slides after distribution. The results were given back within one day of panel distribution. Among these microscopy centers (MCs), 2 (6.25%) could correctly detect negative and positive slides, 2 (6.25%) centers misread negative slides as 1+ to 3+, 18 (56.25%) centers misread 1+ to 3+ as negative; 20 (62.5%) centers misread scanty as negative; 5 (16.6%) centers misread negative as scanty and 25 (78.25%) centers had problems in grading the positive smears and quantification errors (Table 2). Thirteen (40.6%) of the 32 laboratories reported back unsatisfactory (below acceptable score < 80%) results and the remaining 19 (59.4%) had acceptable performance (passing score). However, 8 (25%) of MCs have reported major errors (9 HFN and 1 HFP), 26 (81.25%) MCs committed minor errors (33 LFN, 4 LFP and 49 QE) where as 7 participants MCs (21.9%) committed both major and minor errors with (9 HFN, 13 LFP and 20 QE). One higher clinic MC scored unsatisfactory (score of 50%) with major and minor errors (3 HFN, 1 LFN, and 3 QE). The total score of MCs ranges from 50 to 100%. Two MCs scored 100%; six scored 90%. About 131 (40.9%) of panel slides were incorrectly interpreted by MCs. HFN error observed in 7 (2.2%), LFP in 4 (1.25%), HFP in 1 (0.3%), QE in 71 (22.2%) and LFN errors in 46 (14.4%) of panels slides. Over all agreement was very good with kappa value of 0.87, whereas 131 (40.9%) error committed by all health facilities, 2 (6.6%), 90 (28.3%), 8 (2.5%) and 3 (0.9%) were committed by hospitals, higher clinics, health centers and diagnostic laboratory respectively. However there was no statistically significant differences in errors types and health facilities (p value > 0.05) (Table 3). There was no statistically difference in the frequency of errors and panel slides types (x2 = P value > 0.05). The overall sensitivity, specificity, PPV and NPV of panel reading by microscopy centers were 89%, 96%, 96%, and 90% respectively (Table 1). Both hospital laboratory and NRL were agreed on 38 (63.3%) positive and 12 (20%) negative readings but disagreed on the remaining 10 (16.7%) of slides which comprises 10 (16.7%) FN and 0 FP results, this resulted in sensitivity, specificity, PPV, NPV and accuracy of 79.2%, 100%, 100%, 54.5% and 83.3% respectively having a acceptable agreement with kappa value of 0.61. This showed that more performance of hospital MCs than higher clinics but less than those of health centers and diagnostic laboratories. Reading of health centers and NRL were agreed on 12 (60%) positive and 4 (20%) negative readings but disagreed on the remaining 4 (20%) of slides which comprises 0 FN and 4 (20%) FP results, with sensitivity, specificity, PPV, NPV and accuracy of 100%, 50%, 75%, 50% & 60% respectively with Kappa value of 0.78 showing substantial agreement. Reading of diagnostic laboratory and NRL were agreed on 8 (80%) positive and 2 (20%) negative readings without any discordant results with 100% sensitivity, specificity, PPV, NPV, accuracy and Kappa value of 1.0, which is a perfect agreement.

**Table 2:** Performance of AFB microscopy centers on panel slides in PHF in Addis Ababa 2014 (n, 32)

Health institution		Major errors		Minor error			Total errors
		HFN N (%)	HFP N (%)	LFN N (%)	LFP N (%)	QE N (%)	Major + Minor
Hospitals	On DOT(n,5)	0	0	7 (2.2)	0	6 (1.9)	13 (4%)
	Not on DOT	0	0	2 (0.6)	0	5 (1.6)	7 (2.2%)
Higher clinics	DOT	2 (0.6)	0	10 (3.1)	1 (0.3)	21 (6.6)	34 (10.6%)
	Not on DOT	6 (1.9)	1(0.5)	24 (7.5)	3 (0.9)	33 (10.3)	67 (20.9%)
Health Center	DOT	1(0.5)	0	0	0	3 (0.9)	4 (1.3%)
	Not on DOT	0	0	3 (0.9)	0	1(0.5)	4 (1.3%)
Diagnostic laboratory		0	0	0	0	2	2 (0.6%)

**Table 3:** Performance level of microscopy centers in Addis Ababa, 2014 (n=33)

Lab Code	HFN	HFP	LFN	LFP	QE	Total error	Total scores
LAB-01	0	0	4	0	2	6	70
LAB-02	3	0	1	0	3	7	50
LAB-03	0	0	3	0	1	4	80
LAB-04	0	0	0	1	2	3	85
LAB-05	0	0	2	0	2	4	80
LAB-06	0	0	1	0	2	3	85
LAB-07	1	0	2	0	4	7	60
LAB-08	0	0	0	0	2	2	90
LAB-09	1	0	2	0	4	7	60
LAB-10	0	0	0	0	5	5	75
LAB-11	0	0	1	0	2	3	85
LAB-12	0	0	3	0	2	5	75
LAB-13	0	0	0	0	0	0	100
LAB-14	0	0	0	0	3	3	85
LAB-15	0	0	0	0	0	0	100
LAB-16	0	0	3	0	2	5	75
LAB-17	0	0	3	1	1	5	75
LAB-19	0	0	3	0	2	5	75
LAB-20	0	0	1	0	3	4	80
LAB-21	1	1	2	0	4	8	50
LAB-22	0	0	0	0	2	2	90
LAB-23	0	0	1	0	1	2	90
LAB-24	0	0	1	1	2	4	80
LAB-25	0	0	0	0	2	2	90
LAB-26	0	0	0	0	2	2	90
LAB-27	0	0	1	0	3	4	80
LAB-28	0	0	2	0	4	6	70
LAB-29	1	0	0	0	3	4	75
LAB-30	1	0	3	0	2	6	65
LAB-31	0	0	3	0	1	4	80
LAB-32	1	0	3	0	0	4	75
LAB-33	0	0	1	1	3	5	75
Total	9	1	46	4	71	131	2442
**Average**	**2.8**	**0.3**	**14.4**	**1.3**	**22.2**	**40.9**	**75.6**

### Blinded re checking

**Figure 1:**
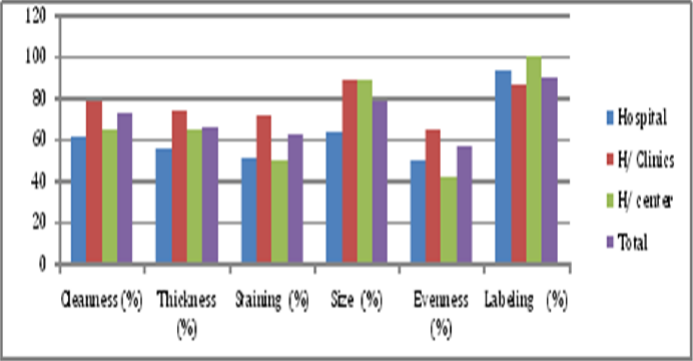
Smear quality of MCs for random blinded rechecked slides in Addis Ababa, 2014 (n, 14)

Among the 33 participant laboratories, blinded rechecking was conducted only in 14 (42.4%) MCs, of them; 5 (35.7 5) hospital, 8 (57.1%) higher clinics and 1 (7.1%) health center. The remaining 19 (57.6%) do not store slides and we could not conduct the blinded rechecking. From 283 random blindly selected and rechecked slides, 14.1% and 85.7% were reported to be positive and negative by microscopy centers and 16.3% and 80.6% were reported as positive and negative by the controller respectively. Out of the 283 re checked slides agreement was observed in 38 (13.4%) positive and 236 (83.4%) negative slides however, disagreed on 9 (3.2%) slides meaning that 7 (2.4%) false negative and 2 (0.7%) false positive results were identified. The sensitivity and specificity of the microscopy centers whether they are on DOT or NOT (n, 14) for blinded rechecking ranges from 85-90% and 90-100% respectively. In general, the overall sensitivity, specificity, PPV and NPV for blinded rechecking were 88.4%, 99.3%, 92.4% and 98.9% respectively with kappa value of 0.87 showing a very good reading agreement. Smear quality were also assessed during the visual re-examination of the sampled smears classifying them as hospital, higher clinics and health centers with average of 221 (78%) had proper smear size, 188 (66.4%) had proper thickness, 175 (61.8%) had proper staining, 199 (73.3%) had cleanness of smears and 161 (56.9%) had evenness of smear. There was significant difference in slide quality among the 14 MCs (p value <0.05) (Figure 1).

### On-site evaluation

A standardized checklist was used to assess the status of infrastructure, SOP, reagent and equipment, maintenance of microscope, biosafety and waste disposal, training related to AFB quality assessment, data and supply management. Nineteen (57.6%) of laboratory have posted smear & staining procedure and grading chart, but only 9 (27.3%) have arranged AFB slides in slide box as per laboratory register and slide number. Twenty-four (72.7%) examines at least 100 fields before reporting negative results, where as 5 (15.2%) and 31 (93.9%) respectively examines 20-50 and 50-70 fields to report negative results. EQA protocol was available and followed in 6 (18.2%) MCs and slides were collected by RRL for EQA from 7 (21.2%) MCs. Five (15.5%) MCs responded as they participated in panel testing, only 2 (6.06%) MCs use control smears at least once a week by preparing positive and negative slides for reagent quality control. Nine (27.3%) MCs use standardized laboratory registration and only 11 (33.3%) MCs and 12 (36.4%) MCs have standardized request form and consistent reporting with complete registration book respectively. Concerning the supply chain managements, only 9 MCs (27.3%) and 11 MCs (33.3%) have inventory system and plan their supplies respectively, while 30 MCs (90.9%) obtained their reagents through direct purchase while the remaining were supplied through Addis Ababa Health regional laboratory and distributed by pharmacist and laboratory professionals. No laboratory services were interrupted due supply problems and no facility prepares reagents in its laboratory. Among the participant facilities, 14 (42.4%) were on DOT program on which on-site evaluation was performed for standards of infrastructure, EQA, internal quality control, staining reagents, safety and waste disposal practices, SOPs, training status, microscope maintenance, data management and inventory management. The results obtained from panel testing, blinded rechecking and the onsite evaluation checklist is almost similar and consistent as overall proficiency result shows 75.6% whose reading agreement was very good with kappa value of 0.87 (Table 2). Similarly, in blinded rechecking, the result showed (3.2%) discordant readings with kappa value of 0.87. The reading agreement between the microcopy center and controller is almost perfect. proficiency testing & blinded rechecking was almost consistent with results from the onsite evaluation with poor usage of posted smear preparation 19 (57.6%); staining procedure & grading chart, while 24 (72.7%) examines at least 100 fields before reporting negative slides, where as 5 (15.2%) and 31 (93.9%) respectively examines 20-50 and 50-70 fields to report negative results. Together with these, the performance quality of the microscopy center was compromised with the availability and implementation of EQA protocol.

## Discussion

An isolated occurrence could often be considered to represent an administrative error such as erroneous recording or mislabeling of the slide, 0.6% of smears had HFP results in one of the 32 laboratories indicating major error and the remaining sites had zero incidence of HFP. An HFN, especially with 1+, could be due to quality of staining and unequal distribution of AFB in the smear. Other reasons include lack of technical knowledge in identifying AFB, poorly maintained microscopes, work overload, carelessness in reading, or not reading the slide at all. Low incidence of HFP (less than 2%) reported from the study sites, similar in America. Whereas administrative & technical issues may also cause FN results, specimen handling and registration or smear preparation, stain formulation, staining technique, microscope performance, and smear examination are some of the factors caused by technical aspects of the laboratory [9]. Slides with low numbers of AFB have higher error rate to detect (46 LFN in 22 sites) because they did not read all fields, or due to the high turnover therefore, the outcome is a false negative result that is in agreement with other studies where the factor most strongly associated with PT performance [10]. This is significant error as patients with paucibacillary disease could give negative results in AFB microscopy and will not receive treatment, resulting in further community spread and failure in diagnosis of PTB. Similar study in Haiti shows occurrence of predominant FN errors that might be due to lack following proper EQA protocol. Our finding is also similar to study in America by Pan American health organization shows FP is caused by specimen container, labeling and patient identification. Administrative or technical issues, specimen handling, might cause false negative results and registration or smear preparation, stain formulation, staining technique, microscope performance, and smear examination are some of the factors caused by technical aspects of the laboratory [11]. Majority of laboratories scored which is below acceptable performance of 80% 14 (43.35%). Hospital laboratories were slightly better than higher clinic laboratories with (x2= p value >0.05) showing no statistical association between hospital laboratories & higher clinic laboratories. The most likely explanation for this performance might be in working environment like better supply management & staff composition among health facilities. Similar in Thimphu, Bhutan & Ethiopia where AFB microscopy quality was 21.1 to 23.1% laboratories and found to be unacceptable, with problem of reading slides properly & unsatisfactory reporting (below acceptable score < 80%) results, reporting both major & minor errors [12].

Incorrectly interpreted panel slides were 131 (40.9%): HFN 7 (2.2%), LFP in 4 (1.25%), HFP in 1 (0.3%), QE in 71 (22.2%) and LFN errors in 46 (14.4%) of panel. Over all agreement was very good with kappa value of 0.87. Among 131 (40.9%) error committed by all health facilities, 2 (6.6%), 90 (28.3%), 8 (2.5%) and 3 (0.9%) were committed by hospitals, higher clinics, health centers and diagnostic laboratory respectively, similar in Karachi hospital where 2 (28.6%) correctly classified; this might be due to poor management, capacity building and rigorous monitoring of standards [13]. There were major errors in 3 (14.3%) laboratories where as minor errors in 12 (57.1%) laboratories. Errors were encountered on 23 (11.0%) of the smears. In contrast to our findings, a panel test done in Nepal, 1 (11.1%) had QE (minor) while no any major error encountered [14]. The possible causes were competency of professionals in identifying AFB, work overload, lack of trainings on AFB training, failure to filter carbol fuchsin regularly, checking sputum quality, consistent result with study Ethiopia where false reading was 3.2% and 74%. Therefore, this could be due to failure to read adequate number of microscopy field, staining and reading problems [15]. Comparison of the results of the microscopy centers with the controller showed similar in positive and negative findings 38 (13.4%) and 236 (83.4%) respectively. Considering the reading of the controller as a true value, there was 9 (3.2%) discordant slides (2 FP and 7 FN) with 99.3% specificity and 84.4% sensitivity. There was no disagreement between microscopy centers and controller having overall agreement of 97.5% (X2= p-value>0.05). This was similar in Nigeria showing with increased concordance rate of FP and FN rates of microscopy results [16]. From 283 random blindly selected slides, 14.1% and 85.7% were reported to be positive and negative for AFB by microscopy centers but 16.3% and 80.6% were reported as positive and negative by the controller respectively with (X2= p-value <0.05) having a statistically significant difference between the microscopy center and the controller, comparable with Kinshasa 77 (10.4%) discrepant results. In addition, 67 (87%) of these discrepant results were attributed to the peripheral laboratory [17].

Standard laboratory request and reporting form were found in 11 (33.3%) deficient in result generation and causing in data management for surveillance and other planning purpose, similar to study done in California [18]. Only 2 (6.06%) use control smears at least once a week, 9 (27.3%) use standardized laboratory registration whereas 12 (36.4%) report consistently with complete registration book, similar to Pan American health organization that shows administrative issues like specimen container, transport condition, specimen quality, patient identification, specimen labeling, handling, patient identification and registration or smearing, stain formulation, staining technique, microscope performance, and smear examination are some of the factors that compromise quality laboratory service, 6 (18.2%) have separate area of AFB specimen receipt and smear preparation. In addition, 15 (45.5%) have well ventilated windows while 29 (87.8%) have regular water supply but most institutions 31 (93.95) and 30 (90.9%) have permanent electric power with backup generator respectively. Likewise, 19 (57.6%) of laboratory have posted smear preparation, staining procedure and grading chart, but only 9 (27.3%) have arranged slides in slide box as per lab register and slide number. Twenty-four (72.7%) examines at least 100 fields before reporting negative, where as 5 (15.2%) and 31 (93.9%) respectively examines 20-50 and 50-70 fields to report negative which is similar to study in Indonesia showing 9 to 53% of tuberculosis cases and 4-18% of sputum smear positive cases in hospitals were not served with standardized diagnosis [19]. Only one laboratory 1 (3.0%) filters carbol fuchsin before staining and 10 (30.3%) filters monthly but 22 (66.7%) of them do not filter at all, 28 (84.8%) use functional binocular microscope and EQA protocol is available and followed in 6 (18.2%) and slides are collected by RRL for EQA from 7 (21.2%). This is also similar to study by WHO in resource-poor countries in which many smear microscopy laboratories are single roomed and understaffed with poorly maintained microscopes, and some of these laboratories lack consistent sources of electricity and clean water. There are few opportunities for the training of staff and little staff capacity to handle high volume workloads [20].

## Conclusion

Proficiency test score of 100%, 80-95% and 50-75% was performed by 2, 15 and 15 laboratories, respectively, since microscopy centers scored a panel test score of 75.5% which is below acceptable 80%. Out of 283 randomly selected slides, the overall, false reading for blinded rechecking was 3.9% with overall agreement of 97.5% and sensitivity of 88.4% and specificity of 99.3%. On-site evaluation indicated poor supply and usages of infrastructure, SOPs, reagent quality, equipment maintenance, data management, and training issues. The results obtained from panel testing, blinded rechecking and the onsite evaluation checklist is almost similar and consistent as overall proficiency result shows 75.6% whose reading agreement was very good with kappa value of 0.87; similarly, in blinded rechecking, the result showed 3.2% discordant readings with kappa value of 0.87. Thus, the reading agreement between the microcopy center and controller was almost perfect. Together with these, the qualities of the microscopy centers depicted compromised findings with the availability and implementation of EQA protocol.

### What is known about this topic

- AFB smear microscopy provides visual evidence of TB bacterial burden and used for routine diagnosis of pulmonary tuberculosis.
- AFB smear microscopy is a commonly performed method in resource limited countries.
- EQA is a method performed to assess the performance of laboratory personnel through the proficiency testing, random blinded re checking and onsite evaluation checklist.

### What this study adds

- Generated data on quality performance of TB laboratory in private health facilities which didn't addressed previously in the capital of Ethiopia.
- It identified gaps of microscopy centers concerning EQA from the private health facilities point of view that further facilitate accreditation process.
- Determined overall situation of the AFB microscopy centers.
